# Application of Analytical Chemistry to Foods and Food Technology

**DOI:** 10.3390/foods9091296

**Published:** 2020-09-15

**Authors:** Daniele Naviglio, Monica Gallo

**Affiliations:** 1 Department of Chemical Sciences, University of Naples Federico II, via Cintia, 21, 80126 Naples, Italy; 2 Department of Molecular Medicine and Medical Biotechnology, University of Naples Federico II, via Pansini, 5, 80131 Naples, Italy

**Keywords:** antioxidants, bioactive compounds, functional foods, gas chromatography, health effects, liquid chromatography (HPLC), mass spectrometry, nutraceuticals, phytochemicals, solid-liquid extraction techniques

## Abstract

Foods are a mixture of substances capable of supplying the human body with nutrients, which, once metabolized, are used mainly for the production of energy, heat, replenishment, and growth material for organs and tissues, ensuring the normal performance of vital functions necessary for growth of the human body. Therefore, the study of the chemical composition of foods and the properties of their constituents helps to define their nutritional and commodity values. Furthermore, it allows for evaluation of the chemical modifications that the constituents of the food undergo following the treatments (Food Technology) to which they are subjected. Analytical chemistry is the branch of chemistry based on the qualitative and quantitative determination of compounds present in a sample under examination. Therefore, through its application, it is possible to determine the quality of a product and/or its nutritional value, reveal adulterations, identify the presence of xenobiotic substances potentially harmful to human health (heavy metals, IPA, pharmaceuticals, etc.). Furthermore, some foods, in particular those of plant origin, contain numerous substances, secondary metabolites, with huge beneficial effects for human health. These functional components can be taken both through a correct diet, but also obtained from different food matrices by technological or biotechnological processes for the formulation of both functional foods and/or nutraceutical products. This Special Issue brings together 10 original studies and two comprehensive reviews on the above topics, in particular: (i) processes of extraction, identification, and characterization of biologically active compounds from different food matrices, (ii) overview of the main techniques applied for the determination of food colors, (iii) newer and greener solid-liquid extraction techniques.

Two centuries ago, the application of Analytical Chemistry to the study of the composition of food gave rise to a new science called Bromatology (from the Greek βρῶμα, which means food). This science, currently referred to as Food Chemistry, can be considered as a branch of chemistry that deals with the study of food, deepening the aspects related to the qualitative and quantitative characterization of its main components (lipids, proteins, carbohydrates, vitamins, and minerals). On the other hand, food products can be consumed as such or subjected to treatments including conservation and transformation technologies, with all the resulting consequences due to the potential lowering in quality of final products [1]. Therefore, the enormous growth of the food industry over the last fifty years has broadened the scope of analytical chemistry not only to food, but also to food technology, which is fundamental for increasing the production of a large gamma of foods.

Furthermore, various scientific evidence has now definitively demonstrated the positive role that some nutritional factors, such as vegetable fibers, antioxidant compounds, particular classes of lipids, bioactive peptides, and so on, present in food matrices, especially of vegetable origin, can play in the prevention of widespread chronic and degenerative diseases (cardiovascular diseases, neoplasms, metabolic syndrome). More specifically, a high consumption of fruit and vegetables has been associated with a lower risk of cardiovascular disease, as well as of some types of neoplasms [2].

In addition, in recent times, even in the food sector, the concept of acircular economy is becoming more and more widespread, i.e., a system based on the ability to Reuse, Recover and Recycle (Three R) waste materials from the various production phases, or even on preventing them. As is known, the production of waste contributes to air, water, and soil pollution, as well as to climate change and the loss of biodiversity. However, it must be considered that some agri-food waste, even after use, retains a mixture of substances that are not removed. Therefore, the current green concept foresees the creation and research of new ways of extracting valuable compounds from plants, herbs, algae, other organisms, but also from waste material, in order to promote sustainable growth of the world population. Consequently, through appropriate technologies, it is possible to recover bioactive compounds to be used in various sectors [3,4].

Starting from the above premises, in recent years, numerous researches have been carried out concerning the identification of substances with beneficial action from various matrices, especially of vegetable origin, in order to evaluate their use in various sectors, such as pharmaceutical, cosmetic, herbal, and food. These studies foresee that the matrices under examination are subjected to extraction processes, with subsequent identification and characterization of molecules of nutraceutical interest. The structural characterization of these molecules is obtained using classical biochemical methods, which, more recently, are usually integrated with advanced techniques, by means of proteomic and metabolomic approaches based on chromatographic, electrophoretic, mass spectrometry, and nuclear magnetic resonance technologies. The same extraction, identification, and characterization procedures are also applied to processed products and/or other systems of interest.

Below is a brief review of the articles made by international research groups that have allowed the realization of this Special Issue. The research areas range from studies on biologically active compounds obtained from different food matrices to the most recent extraction techniques, from food technologies proper up to quality control and food safety, using some of the most innovative approaches available today. In particular, they concern the extraction, identification and characterization of molecules of nutraceutical interest, especially of plant origin by means of proteomic and metabolomic approaches based on chromatographic, electrophoretic, and spectroscopic technologies.

Curcuma is a perennial herb of Indonesian origin. The noble part of the plant is the rhizome. The latter contains curcumin and curcuminoids, active ingredients of turmeric, much studied for their beneficial effects. Binello et al. (2020) describe the extraction of curcuminoids from *Curcuma longa* L. rhizomes by ultrasound-assisted extraction (UAE). The results show, for the first time, an analytical evaluation of the stability of curcuminoids under sonication in different solvents [5].

Kobbah is an oriental dish consisting of ground bulgur (grain-based food) mixed with ground beef. Typically, the dish is prepared in the form of balls filled with cooked minced meat, onions, nuts, and spices, cooked by frying. Al-Asmar et al. (2020) investigated the influence of different hydrocolloid-based coatings (containing pectin and chickpea flour prepared in the presence or absence of nanoparticles and/or transglutaminase) on the content of acrylamide, water, oil, digestibility and color of fried kobbah. The physico-chemical properties of different coating solutions were also evaluated. The results show that the best coating solution that significantly reduced acrylamide was the one made from pectin, confirming that increasing the water content within the fried food by coating is an effective way to mitigate the formation of acrylamide and the oil content [6].

In a paper by Wang et al. (2020), quantitative analysis of spectinomycin and lincomycin in poultry egg samples was performed by accelerated solvent extraction (ASE) coupled with gas chromatography tandem mass spectrometry (GC-MS/MS). The results show that the proposed method is faster, requires fewer reagents and more samples can be processed at a time, compared to conventional extraction methods [7].

A paper by Hirondart et al. (2020) reports the comparison of two extraction techniques, such as conventional Soxhlet extraction and pressurized liquid extraction (PLE) to determine the initial composition of the key antioxidants contained in rosemary leaves. The data obtained show that there are no significant differences between the two procedures in terms of extraction, but PLE is a quick, clean, and environmentally friendly extraction technique [8].

*Colchicum triphyllum* is a little-known Turkish cultivar belonging to the *Colchicaceae*. Senizza et al. (2020) evaluated the antioxidant and enzyme inhibitory effects in vitro of extracts of flowers, tubers and leaves of this cultivar, obtained by different extraction methods, such as maceration, infusion, and Soxhlet. The interesting data obtained show the potential of *C. triphhyllum* extracts in food and pharmaceutical applications [9].

The presence of lead in the environment and in the food chain represents a serious pollution problem. In fact, multiple health effects are associated with exposure to heavy metals, with different degrees of severity and conditions: kidney and bone problems, neurobehavioral and developmental disorders, high blood pressure and, potentially, even lung cancer. In this context, immunoassays for the quantitative measurement of environmental heavy metals offer numerous advantages over other traditional methods. Therefore, the methods developed by Xu et al. (2020) through the use of ELISA and chemiluminescent enzyme immunoassay allowed the determination of trace lead (II) in various samples with high sensitivity, simplicity, and accuracy [10].

Liu et al. (2020) investigated the characteristic aroma components of five Chinese mango varieties using headspace solid phase microextraction (HS-SPME) coupled with gas chromatography-mass spectrometry-gas chromatography-olfactometry (GC-MS-O) techniques. Based on these techniques, five main types of substances have been detected, including alcohols, terpenes, esters, aldehydes, and ketones, which are responsible for their special flavor [11].

Literature data report that the fruits of the oleaster (*Elaeagnus angustifolia*) as well as being used as food, contain components with antinociceptive and anti-inflammatory effects. However, narrow-leaved olive fruits have different geographic origins that vary in chemical and physical properties and differ in their nutritional and commercial values. In a study by Gao et al. (2019), near-infrared hyperspectral imaging was used to identify the geographic origins of dry narrow-leaved olive fruits with machine learning methods. The overall results illustrated that this approach could be used to trace the geographical origins of oleaster fruits [12].

Tsochatzis et al. (2019) developed and validated an Ultra High Performance Hydrophilic Liquid Chromatography (UHPLC) (HILIC) tandem mass spectrometry (MS) method for the quantification of amino acids in organic and conventional flour samples with different extraction rates. The results showed significant differences in the amino acid profiles of the flours studied [13].

Hsian-tsao (*Platostoma palustre* Blume) is a species of plant belonging to the genus Platostoma of the mint family. In a paper by Kung et al. (2019) analysis of volatile components present in eight varieties of Hsian-tsao was performed using headspace solid phase microextraction (HS-SPME) and simultaneous distillation-extraction (SDE) coupled with gas chromatography (GC) and gas chromatography-mass spectrometry (GC/MS). The analysis of the results obtained made it possible to identify 120 volatile components. In particular, SDE was able to detect more components, while the HS-SPME analysis was more convenient [14].

Food colorants are natural or artificial substances that give color to a food or restore its original color. Therefore, they are classifiable as food additives and are widely used in the food industry. However, in some cases, their presence can pose risks to human health. Consequently, their determination is extremely important. In a review by Ntrallou et al. (2020) an overview of the main techniques applied for the determination of food colorants and sample preparation procedures that strongly depend on the food matrix is presented over the last 10 years [15].

The extraction process represents a very important step in obtaining compounds of interest from a certain matrix. Alongside the conventional extraction techniques still used, there are currently so-called green techniques. In fact, in order for the extraction processes to be defined sustainable, it is important to make use of technologies with high energy efficiency, but based on low environmental impact solvents. In the review by Naviglio et al. (2020) a comparison was made between the most recent solid liquid extraction techniques with the rapid dynamic solid liquid extraction (RSLDE) as a valid alternative to those examined. In particular, in the RSLDE, the extraction takes place for the generation of a negative pressure gradient from the inside to the outside of the solid matrix, so it can be carried out at room temperature or even sub-environment [16].

In conclusion, the results obtained from the various studies presented can contribute significantly to the identification of new bioactive molecules and to the description of the structural and functional properties of the various bioactive compounds, in order to consider their possible use in various sectors. On the other hand, the research areas range from studies on the extraction, identification, and characterization of bioactive compounds, from food technologies proper up to quality control and food safety, using some of the most innovative approaches available today. Therefore, the multidisciplinary approach contained in this Special Issue, in terms of both knowledge and technologies, can constitute a reference point for the study and evaluation of the effect of foods, natural substances, and nutraceuticals on social wellness.
